# Calcium-Sensing Receptor Antagonist NPS 2143 Restores Amyloid Precursor Protein Physiological Non-Amyloidogenic Processing in Aβ-Exposed Adult Human Astrocytes

**DOI:** 10.1038/s41598-017-01215-3

**Published:** 2017-04-28

**Authors:** Anna Chiarini, Ubaldo Armato, Daisong Liu, Ilaria Dal Prà

**Affiliations:** 1 Human Histology & Embryology Unit, Medical School, University of Verona, Verona, Venetia Italy; 2 The Third Xiangya Hospital of Central South University, Department of Plastic Surgery, Changsha, Hunan China

## Abstract

Physiological non-amyloidogenic processing (NAP) of amyloid precursor holoprotein (hAPP) by α-secretases (e.g., ADAM10) extracellularly sheds neurotrophic/neuroprotective soluble (s)APPα and precludes amyloid-β peptides (Aβs) production via β-secretase amyloidogenic processing (AP). Evidence exists that Aβs interact with calcium-sensing receptors (CaSRs) in human astrocytes and neurons, driving the overrelease of toxic Aβ_42_/Aβ_42_-os (oligomers), which is completely blocked by CaSR antagonist (calcilytic) NPS 2143. Here, we investigated the mechanisms underlying NPS 2143 beneficial effects in human astrocytes. Moreover, because Alzheimer’s disease (AD) involves neuroinflammation, we examined whether NPS 2143 remained beneficial when both fibrillary (f)Aβ_25–35_ and a microglial cytokine mixture (CMT) were present. Thus, hAPP NAP prevailed over AP in untreated astrocytes, which extracellularly shed all synthesized sAPPα while secreting basal Aβ_40/42_ amounts. Conversely, fAβ_25–35_ alone dramatically reduced sAPPα extracellular shedding while driving Aβ_42_/Aβ_42_-os oversecretion that CMT accelerated but not increased, despite a concurring hAPP overexpression. NPS 2143 promoted hAPP and ADAM10 translocation to the plasma membrane, thereby restoring sAPPα extracellular shedding and fully suppressing any Aβ_42_/Aβ_42_-os oversecretion, but left hAPP expression unaffected. Therefore, as anti-AD therapeutics calcilytics support neuronal viability by safeguarding astrocytes neurotrophic/neuroprotective sAPPα shedding, suppressing neurons and astrocytes Aβ_42_/Aβ_42_-os build-up/secretion, and remaining effective even under AD-typical neuroinflammatory conditions.

## Introduction

Alzheimer’s disease (AD) causes dementia in tens of millions of people worldwide^1^. The typical markers of AD are senile plaques, i.e., accumulations of amyloid-β peptides (Aβs) in the neuropil; neurofibrillary tangles (NFTs), i.e., insoluble clusters of hyperphosphorylated Tau proteins inside neurons; the loss of synaptic contacts; neurotoxicity; increased cell death in neurons and oligodendrocytes; activated astrocytes and microglia; chronic neuroinflammation; cerebrovascular damage; and blood-brain barrier dysfunction^2–4^. These markers are present in extended cortical areas up to fifteen years before the onset of any memory or cognition decline^2, 5, 6^.

Amyloid precursor holoprotein (hAPP)’s pathophysiological relevance in AD is notorious^7, 8^. Human hAPP and its mammalian homologues AP-like protein 1 (APLP1) and 2 (APLP2) are integral transmembrane proteins that promote cell adhesion by homo- or hetero-dimerization or functioning as receptors^9^. Their manifold physiological activities include the modulation of cell survival, axonal growth, synaptogenesis, synaptic plasticity, and neuronal excitability^7, 8, 10^. hAPP is synthesized in the endoplasmic reticulum (ER), post-translationally modified by N- and O-linked glycosylation, and transported along the secretory pathway to the cell surface^11^.

Importantly, hAPP undergoes proteolysis via two pathways: amyloidogenic processing (AP) and non-amyloidogenic processing (NAP). In AP, BACE (β-site APP cleaving enzyme)1/β-secretase, a membrane-bound aspartyl protease, cleaves the N-terminus of the hAPP Aβ domain in the *trans*-Golgi network (TGN) and, following endocytosis, in the endosomal/lysosomal system^11–15^, thereby releasing (shedding) a pro-apoptotic soluble (s)APPβ ectodomain^14, 15^. The membrane-bound γ-secretase complex, comprising presenilins 1 (PS1) and 2 (PS2), cleaves the C99 or CTFβ (99 amino acid C-terminal fragment) of membrane-bound hAPP, producing (*i*) Aβs and (*ii*) the hAPP intracellular domain (AICD) peptide, which might play a transcriptional role^16–19^. Therefore, mutations in the genes encoding hAPP, PS1 or PS2 drive early-onset familial AD (EOFAD; <3% cases) by driving the surplus production/release of Aβs, which then cluster as variously sized toxic oligomers (Aβ-os) or polymers (fibrils)^19^. Conversely, the aetiologic factors underlying the prevailing (>95% cases) late-onset (sporadic) AD (LOAD) cases are likely manifold, slow-acting, and less defined than those underlying EOFAD. Only heterozygous or homozygous apolipoprotein E (APOE) ε4 alleles and TREM-2 mutations, chiefly R47H, aid the onset and progression of LOAD^20^. Therefore, various vascular and metabolic shortcomings interact to hinder the brain’s clearance of the Aβs that accumulate in the neuropil^2–4, 21^. However, even in LOAD, neuropil-clogging Aβs drive, via Aβ•CaSR (calcium-sensing receptor) signalling, increased AP of hAPP, causing the surplus production/release of Aβ_42_/Aβ_42_-os from both astrocytes and neurons. Such Aβs devastatingly spread from the hippocampus to a wide range of upper cortical areas^22–24^.

Under physiological conditions, hAPP NAP typically prevails: hAPP is mostly cleaved between amino acids 612 and 613 within the Aβ domain by α-secretase, which impedes any Aβ production^7, 19^. Physiologically, ADAM10 (a disintegrin and metalloprotease domain-containing protein 10) is the principal hAPP α-secretase in neurons^25^, which acts mainly at the cell surface and within the TGN^26, 27^ and sheds the soluble (s)APPα ectodomain from hAPP^25, 28^.

sAPPα acts as a neurotrophic and neuroprotective compound in brain ischaemic, traumatic, and excitotoxic injuries^29, 30^. Interestingly, sAPPα levels decrease in the cerebrospinal fluids of AD patients^31^. This decrease implies a partial loss of sAPPα activity, resulting in reduced plasticity, connectivity, and synaptic signalling, all of which favour the demise of neurons that occurs in AD^10, 31^. The ectodomains of both membrane-bound hAPP and shed sAPPα play neuroprotective roles *in vitro* ^25, 30, 32–37^, although the receptor(s) and signalling pathway(s) involved remain unclear. Moreover, we know little about the complex mechanisms that modulate hAPP NAP and AP balance, particularly in cortical untransformed human neurons and astrocytes; however, hAPP intracellular trafficking, γ-secretase^38^, and α-secretase^27, 39^ are involved. Undoubtedly, clarification of the mechanisms involved would impact our understanding of AD pathophysiology and therapy.

Previously, most studies focused on neurons as the main sites of BACE1/β-secretase expression^40, 41^ and Aβ production in mouse brains^3, 42, 43^. Activated microglia are the second most investigated brain cell type, as they engulf fibrillar and soluble Aβs, promote Aβ clumping inside senile plaques, secrete proinflammatory cytokines, and overrelease toxic nitric oxide (NO) and reactive oxygen species (ROS)^3, 4, 44^. Conversely, astrocytes were believed to scavenge, accumulate, proteolyze, and release Aβs, thereby promoting the build-up of glial fibrillary acidic protein (GFAP)-rich senile plaques^45^. However, recent evidence shows that astrocytes also play pivotal roles in the onset and progression of human AD, being more numerous than neurons, establishing extended inter-astrocyte networks via gap junctions, forming teams with client neurons, wrapping tripartite synapses, sheltering neurons from environmental toxins, and exchanging with them physiological and pathological (e.g., Aβs) metabolic compounds^46–48^. Eventually, the benefits of the neuron-sustaining activities of astrocytes vanish when the Aβ-clearing activity becomes too intense^45–49^.

Blasko *et al*.^50^ first reported that human astrocytes produce Aβ_40_ and Aβ_42_ upon exposure to pairs of microglial proinflammatory cytokines, such as interferon (IFN)-γ plus tumour necrosis factor (TNF)-α or interleukin (IL)-1β^44^. Activated cortical astrocytes from neonatal mice also exhibited elevated levels of hAPP, BACE1 activity, and Aβ_40_ secretion when challenged with IFN-γ + TNF-α or sAβ_42_ or fAβ_42_^51^. These findings suggested that neuroinflammation might drive Aβ production/release from human and rodent astrocytes, thus increasing the Aβ load in the brain. Importantly, cortical untransformed adult human astrocytes synthesize, accumulate, and secrete increased amounts of endogenous Aβ_42_/Aβ_42_-os when exposed to exogenous fAβ_25–35_ or sAβ_25–35_^22–24, 52^. Additionally, exogenous Aβs specifically bind plasma membrane CaSRs and activate their manifold signalling, which drives repeated cycles of exogenous Aβs ⇒ endogenous Aβ42 production/secretion in human cortical astrocytes and neurons^22–24, 53^.

CaSRs, which are a family C G-protein-coupled receptors, quickly recognize changes in extracellular Ca^2+^ levels [Ca^2+^]_e_, yet also bind other cations such as polyamines, amino glycoside antibiotics, and Aβs^22–24, 53, 54^. The intracellular domains of CaSRs interact with various G-proteins to modulate a complex set of signalling activities, including (*i*) protein kinases (AKT, PKCs, MAPKs); (*ii*) phospholipases (A2, C, and D); (*iii*) transcription factors (TFs); (*iv*) second messenger production (e.g., cAMP); and (*v*) Ca^2+^ influx via TRPC6-encoded receptor-operated channels^55, 56^. All cells in the central nervous system (CNS) express CaSRs, though expression levels vary by region, with particularly high expression in the hippocampus^57^. In addition, proliferatively quiescent untransformed adult human astrocytes express functional CaSRs more strongly than growing astrocytes independent of [Ca^2+^]_e_ changes^58^. Aside from regulating general Ca^2+^ homeostasis^55^, CaSRs play additional important roles in the CNS^24, 59^.

Altered expression or dysfunction of CaSRs also impacts CNS diseases, such as AD and ischaemia/hypoxia/stroke, by modulating amyloidogenesis, glial activation, outward K^+^ channel fluxes, NO and VEGF-A overproduction, and neuronal death^22–24, 57, 60–63^.

The use of non-tumorigenic cortical adult human astrocytes and postnatal HCN-1A neurons as *in vitro* preclinical models has revealed a novel pathological interaction by which exogenous Aβs bind and activate CaSRs^22–24^. In addition to the transient overexpression of the CaSR, this interaction leads to the surplus production, accumulation and secretion of Aβ_42_-os from both astrocytes and neurons, coupled with increased cell death among the latter. The CaSR agonist NPS R-568 increases Aβ_42_-os release from human cortical astrocytes, mimicking the effect of Aβ_25–35_•CaSR signalling^22–24^. Simultaneously, Aβ-os•CaSR signalling in astrocytes induces the surplus production/release of NO and VEGF-A^58, 61^. These findings suggested that various phenylalkylamines, acting as allosteric CaSR antagonists (calcilytics; e.g., NPS 89636 and NPS 2143) capable of shifting the CaSR response curve to changes in [Ca^2+^]_e_^54, 64^, may be potential AD therapeutics. Calcilytic NPS 2143 suppressed all aforementioned neurotoxic effects elicited by exogenous Aβ_25–35_•CaSR signalling and fully preserved neuronal viability^22–24^.

Given the potential value for AD therapy, in this work, we explored for the first time the mechanisms underlying the beneficial effects of NPS 2143 in human astrocytes, particularly the effects of Aβ•CaSR signalling on the NAP of hAPP and the modulation of such effects by a calcilytic. Moreover, as AD progression in the human brain is coupled with diffuse, chronic neuroinflammation^65^, we sought to determine whether a calcilytic might still antagonize the noxious effects of fAβ_25–35_•CaSR signalling when a microglial cytokine mixture trio (CMT; i.e., IFN-γ, TNF-α, and IL-1β) was also administered^58^. The results reported herein further strengthen our view^22–24^ that calcilytics embody a novel class of anti-AD therapeutics that effectively maintain the physiological shedding of neurotrophic and neuroprotective sAPPα by human astrocytes, as well as fully antagonizing all Aβ_25–35_•CaSR signalling-elicited neurotoxic effects, even in the presence of microglial proinflammatory cytokines.

## Results

The specific involvement of CaSRs in the following studies was shown by the inhibitory effects of a highly selective antagonist, NPS 2143, on the NO release and cAMP levels elicited by various treatments (see Supplementary Information and Fig. S1). The reversemer peptide Aβ_35–25_ was always ineffective (not shown).

### Calcilytic NPS 2143 suppresses Aβ_42_/Aβ_42_-os oversecretion driven by fAβ_25–35_ ± CMT

#### Secreted and intracellular Aβ_42_ levels

**Figure 1:**
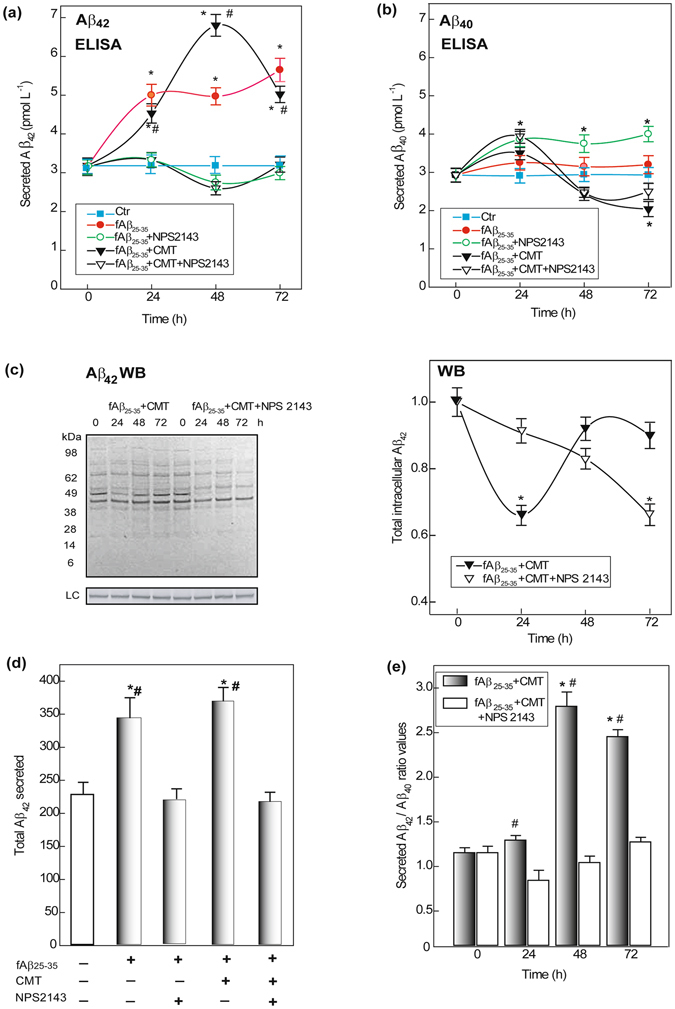
Changes in human astrocytes’ secreted and intracellular amounts of endogenous Aβ_42_ and Aβ_40_. **(a)** Aβ_42_ peptides secreted amounts increase after an exposure to fAβ_25__−35_ ± CMT, but stay unchanged when NPS 2143 is also added. Points on the curves are means ± SEM of 3–5 independent experiments; fAβ_25–35_ + CMT ± NPS 2143: *one-way ANOVA* analysis of the complete data set *F* = 140.805, P < 0.001. *Bonferroni t-test*: comparisons of each treated group *versus* 0-h group (controls): *P < 0.05; pair-wise comparisons between corresponding treatments ± NPS 2143 at each time point: **#**P < 0.05. **(b)** Changes in Aβ_40_ released amounts according to the treatments. Points on the curves are means ± SEM of 3–5 independent experiments; fAβ_25–35_ + CMT ± NPS2143: *one-way ANOVA* analysis of the complete data set *F* = 13.871, P < 0.001. *Bonferroni t-test*: comparisons of each treated group *versus* 0-h group (controls): *P < 0.05. **(c)** *Left panel*. Typical immunoblot revealing the changes in intracellularly accumulated Aβ_42_/Aβ_42_-os versus untreated controls according to treatments. LC, loading control. *Right panel*. Densitometric evaluations of the whole sets of intracellular Aβ_42_ bands at each time point. Points on the curves are means ± SEM of 3 independent experiments, with control values normalized as 1.0. *One-way ANOVA* analysis of the complete data set *F* = 3.933, P < 0.012. *Bonferroni t-test*: comparisons of each treated group *versus* 0-h group (controls): *P < 0.05. **(d)** Alterations in total 0-to-72-h amounts of secreted Aβ_42_ according to treatments. Bars are means ± SEM of 5 independent experiments. *One-way ANOVA* analysis of the complete data set *F* = 13.248, P < 0.001. *Bonferroni t-test*: comparisons of each treated group *versus* 0-h group (controls): *P < 0.05; pair-wise comparisons between corresponding treatments ± NPS 2143: **#**P < 0.05. **(e)** Changes in secreted Aβ_42_/Aβ_40_ ratio values according to treatments in the conditioned growth media of astrocytes. Bars are means ± SEM of 3 independent experiments. *One-way ANOVA* analysis of the complete data set *F* = 73.769, P < 0.001; *Bonferroni t-test*: comparisons of each treated group *versus* 0-h group (controls): *P < 0.05; pair-wise comparisons between corresponding treatments ± NPS 2143 at each time point: **#**P < 0.05.

The amount of endogenous Aβ_42_/Aβ_42_-os released from fAβ_25–35_-exposed human astrocytes rose quickly at first and then more slowly. Co-treatment with CMT hastened the secretion of endogenous Aβ_42_/Aβ_42_-os, which peaked at 48 h (Fig. 1a). However, CMT addition only marginally increased the total amount of Aβ_42_/Aβ_42_-os secreted over 72 h versus fAβ_25–35_ alone (Fig. 1d). NPS 2143 treatment fully suppressed the surplus secretion of endogenous Aβ_42_/Aβ_42_-os caused by fAβ_25–35_ ± CMT, maintaining the total amount of Aβ_42_/Aβ_42_-os secreted from 0 h to 72 h at control levels (Fig. 1a,d).

In contrast, fAβ_25–35_ + CMT treatment transiently decreased intracellular Aβ_42_/Aβ_42_-os levels by 24 h (Fig. 1c). Moreover, the addition of NPS 2143 along with fAβ_25–35_ + CMT elicited a progressive decline in intracellular Aβ_42_/Aβ_42_-os levels (Fig. 1c). Thus, both CMT and NPS 2143, either alone or together, hindered the fAβ_25–35_-driven intracellular build-up of Aβ_42_/Aβ_42_-os. A previous densitometric analysis of specific immunoblot bands showed that between 0 h and 48 h, the total intracellular levels of Aβ_42_/Aβ_42_-os more than tripled in fAβ_25–35_-treated astrocytes versus untreated controls. Conversely, NPS 2143 fully suppressed this fAβ_25–35_-elicited effect at all time-points (not shown; details in Armato *et al*.^22^).

#### Intracellular and secreted Aβ_40_ levels

Adding CMT ± NPS 2143 with Aβ_25–35_ suppressed any surge in intracellular Aβ_40_ levels (data not shown). fAβ_25–35_ + CMT treatment decreased Aβ_40_ secretion only after 72 h. NPS 2143 + fAβ_25–35_ steadily increased Aβ_40_ secretion. However, adding NPS 2143 + fAβ_25–35_ + CMT caused a transient spike in Aβ_40_ secretion after 24 h (Fig. 1b). Therefore, the pattern of Aβ_40_ secretion sharply differed from that of Aβ_42_ in adult human astrocytes (cf. Fig. 1a and b). Previously, we observed a significant fAβ_25–35_-induced intracellular build-up of Aβ_40_, which peaked at 24–48 h and was only marginally affected by NPS 2143 treatment for 48 h; conversely, fAβ_25–35_ alone did not alter the level of Aβ_40_ secretion versus untreated controls (for details, see ref. 22).

#### Secreted Aβ_42_/Aβ_40_ ratios

In the astrocyte-conditioned growth media, Aβ_25–35_ + CMT increased the secreted Aβ_42_/Aβ_40_ ratio into the cytotoxic range, similar to Aβ_25–35_ alone (see for details^22^) (Fig. 1e). Conversely, adding NPS 2143 maintained the secreted Aβ_42_/Aβ_40_ ratios of fAβ_25–35_ + CMT-exposed astrocytes within the non-cytotoxic range observed in the controls (Fig. 1e). Therefore, by suppressing the overrelease of endogenous Aβ42/Aβ42-os in the presence of Aβs + CMT, NPS 2143 further highlighted the potential roles of Aβ•CaSR signalling in AD development.

### NPS 2143 rescues the fAβ_25–35_ ± CMT-induced block in the physiological shedding of sAPPα

Several studies have highlighted the key physiological roles hAPP NAP plays through the shedding of neurotrophic and neuroprotective sAPPα via α-secretase activity^18, 25, 65^. Therefore, we investigated how sAPPα shedding is altered in human astrocytes exposed to fAβ_25–35_ ± CMT ± NPS 2143.

#### Extracellular and intracellular sAPPα levels

**Figure 2:**
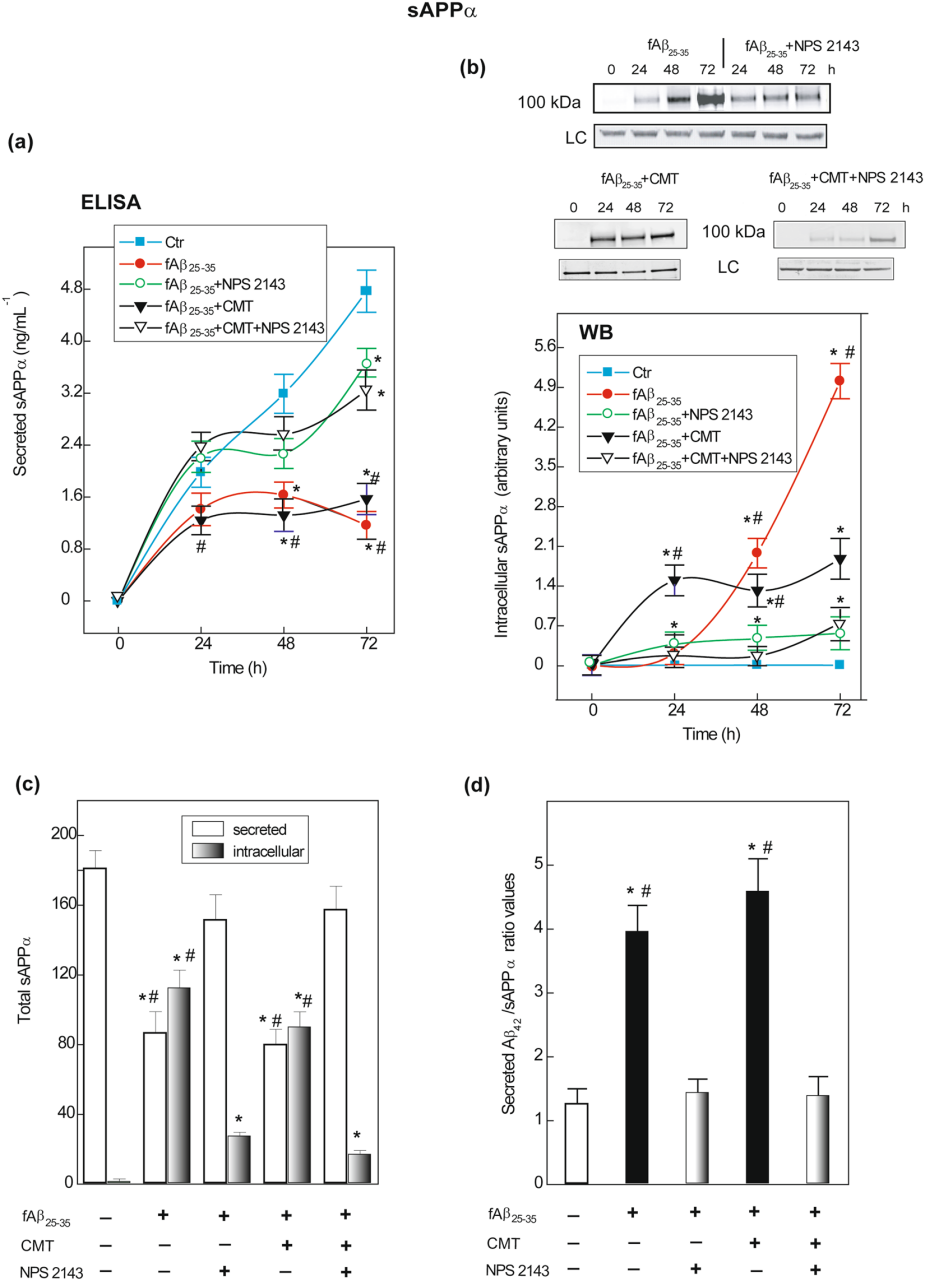
Changes in human astrocytes intracellularly accumulated and extracellularly shed amounts of sAPPα according to treatments. **(a**) Cumulative curves of sAPPα shed into the medium by astrocytes as affected by treatments. Points on the curves are means ± SEM of 5 independent experiments. *One-way ANOVA* analysis of each complete data set: fAβ_25–35_: *F* = 80.877, P < 0.001; fAβ_25–35_ + NPS 2143: *F* = 35.365, P < 0.001; fAβ_25–35_ + CMT: *F* = 50.882, P < 0.001; fAβ_25–35_ + CMT + NPS 2143: *F* = 34.261, P < 0.001. *Bonferroni t-test*: comparisons of each treated group *versus* 0-h group (controls): *P < 0.05; pair-wise comparisons between corresponding treatments ± NPS 2143 at each time point: ^**#**^P < 0.05. **(b**) *Top*. Typical immunoblot revealing the changes in intracellularly accumulated sAPPα according to treatments *versus* controls (0-h). LC, loading control. Blots have been cropped to size for clarity. *Bottom*. Densitometric evaluations of intracellular sAPPα bands. Points on the curves are means ± SEM of 3 independent experiments, with 0-h values normalized as 1.0. *One-way ANOVA* analysis of each complete data set: fAβ_25–35_ ± NPS 2143: *F* = 280.483, P < 0.001; fAβ_25–35_ + CMT ± NPS2143: *F* = 134.239, P < 0.001. *Bonferroni t-test*: comparisons of each treated group *versus* 0-h group (controls): *P < 0.05; pair-wise comparisons between corresponding treatments ± NPS 2143 at each time point: ^**#**^P < 0.05. **(c**) The alterations in total 0-to-72-h amounts of extracellularly shed and intracellular sAPPα according to treatments or no treatment (controls). Bars are means ± SEM of 3–5 independent experiments. *One-way ANOVA* analysis of the (*i*) shed sAPPα complete data set: *F* = 44.056, P < 0.001; (*ii*) intracellular sAPPα complete data set: *F* = 587.435, P < 0.001. *Bonferroni t-test*: comparisons of each treated group *versus* untreated group (controls): *P < 0.05; pair-wise comparisons between corresponding treatments ± NPS 2143 of both intracellular and shed values, respectively: ^**#**^P < 0.05. **(d**) Changes in secreted Aβ_42_/sAPPα ratio values according to treatments or no treatment (controls). Bars are means ± SEM of 5 separate experiments. *One-way ANOVA* analysis of the complete data set *F* = 22.123, P < 0.001. *Bonferroni t-test*: comparisons of each treated group versus 0-h (controls): *P < 0.05; pair-wise comparisons between corresponding treated group versus added NPS 2143 group: ^**#**^P < 0.05.

Untreated (control) astrocytes constitutively shed steady amounts of sAPPα into the medium (Fig. 2a). However, fAβ_25–35_ ± CMT steadily and similarly decreased sAPPα shedding, while simultaneously inducing the oversecretion of endogenous Aβ_42_ (cf. Fig. 1a). Remarkably, adding NPS 2143 to fAβ_25–35_ ± CMT rescued most of the sAPPα shedding (Fig. 2a).

The cleavage of hAPP by ADAM10 is reported to occur at the plasma membrane, the TGN, and in post-TGN vesicles^27^. Hence, we also investigated the intracellular accumulation/retention of sAPPα (Fig. 2b). Between 0 h and 72 h, intracellular sAPPα was undetectable in untreated (control) astrocytes. Conversely, fAβ_25–35_ alone induced a delayed but progressive increase in intra-astrocyte sAPPα accumulation, which was mostly abolished by NPS 2143. fAβ_25–35_ + CMT treatment also increased intra-astrocyte sAPPα accumulation, which was almost completely abolished by NPS 2143. Despite the ongoing increased extracellular shedding of sAPPα (cf. Fig. 2a), modest intracellular sAPPα accumulation was observed by 72 h (1/16 of that evoked by fAβ_25–35_ alone) due to an unknown, Aβ·CaSR signalling-independent mechanism (Fig. 2b).

#### Total changes in sAPPα levels over 72 h

A summary of the total changes in extracellularly shed (*white bars*) and intracellularly accumulated (*black-grey bar*s) sAPPα levels over 72 h (derived from the areas under the respective curves) is shown in Fig. 2c. Unquestionably, fAβ_25–35_ ± CMT strongly increased intracellular sAPPα retention while reducing its extracellular shedding (Fig. 2a) and increased the secretion of Aβ_42_/Aβ_42_-os (Fig. 1a). The Aβ_42_/sAPPα ratio increased between 3.9- and 4.5-fold versus the control in fAβ_25–35_ ± CMT-treated astrocyte media, indicating a robust enhancement of hAPP AP (Fig. 2d). Conversely, adding NPS 2143 to fAβ_25–35_ ± CMT-treated astrocytes maintained the Aβ_42_/sAPPα ratio close to control levels (Fig. 2d). Clearly, by antagonizing Aβ·CaSR signalling, the calcilytic nearly fully restored the physiological, non-amyloidogenic shedding of neurotrophic sAPPα while hindering any Aβ_42_/Aβ_42_-os oversecretion from fAβ_25–35_ ± CMT-treated astrocytes (Figs 2a and 1a).

### NPS 2143 drives plasma membrane translocation of hAPP in fAβ_25–35_ ± CMT-exposed astrocytes

**Figure 3:**
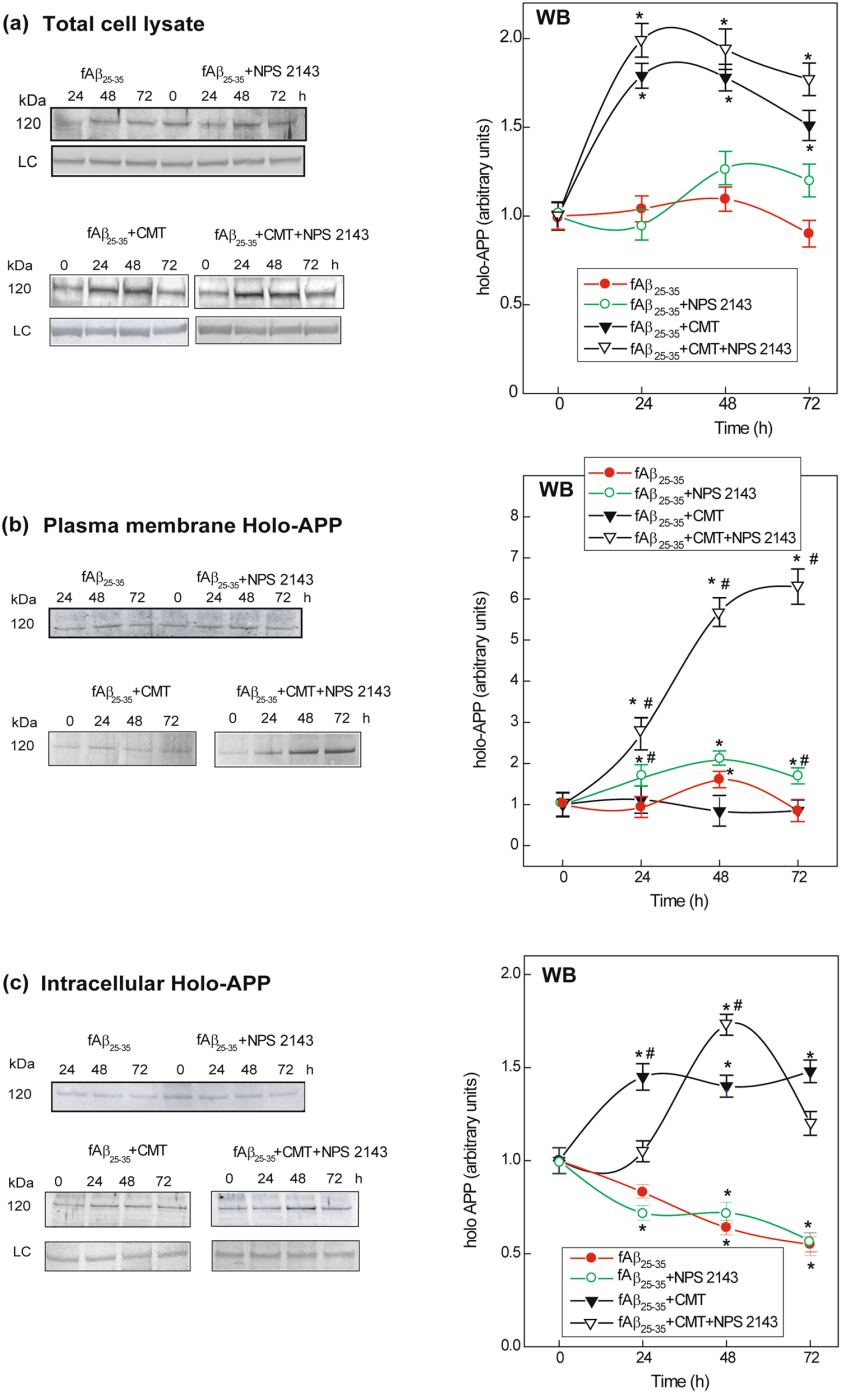
Holo-APP expression and distribution in human untreated and treated astrocytes. **(a**) *Left panel*. Representative immunoblots showing the changes in holo-APP expression according to experimental treatments versus untreated controls in total astrocytes lysates. LC, loading control. In all cases blots have been cropped to size for clarity. *Right panel*. Densitometric evaluations of holo-APP bands for each treatment and time point. Points on the curves are means ± SEM of 3 independent experiments, with 0-h values normalized as 1.0. *One-way ANOVA* analysis of the complete data set: *F* = 8.889, P < 0.001; *Bonferroni t-test* comparisons of each treated group versus 0-h (controls): *P < 0.05. **(b**) *Left panel*. Typical immunoblots showing alterations in plasma membrane hAPP levels (details in *Methods*) according to experimental treatments versus untreated controls. In all cases blots have been cropped to size for clarity. *Right panel*. Densitometric evaluation of holo-APP specific bands for each treatment and time point. Points on the curves are means ± SEM of 3 independent experiments, with 0-h values normalized as 1.0. *One-way ANOVA* analysis of (*i*) fAβ_25–35_ ± NPS 2143 data set: *F* = 14.520, P < 0.001; (*ii*) fAβ_25–35_ + CMT ± NPS 2143 data set: *F* = 240.620, P < 0.001; *Bonferroni t-test:* comparisons of each treated group versus 0-h (controls): *P < 0.05; pair-wise comparisons between corresponding treatments ± NPS 2143 at each time point: ^**#**^P < 0.05. **(c**) *Left panel*. Characteristic immunoblots showing the intracellular holo-APP’s changes versus untreated (0-h) controls according to treatments. LC, loading control. In all cases blots have been cropped to size for clarity. *Right panel*. Densitometric evaluations of specific holo-APP bands for each treatment and time point. Points on the curves are means ± SEM of 3 separate experiments, with 0-h values normalized as 1.0. *One-way ANOVA* analysis of: (*i*) fAβ_25–35_ ± NPS 2143 data set: *F* = 9.800, P < 0.001; (*ii*) fAβ_25–35_ + CMT ± NPS 2143 data set: *F* = 10.710, P < 0.001; *Bonferroni t-test:* comparisons of each treated group versus 0-h (controls): *P < 0.05; pair-wise comparisons between corresponding treatments ± NPS 2143 at each time point: ^**#**^P < 0.05.

hAPP was discretely expressed in total protein lysates of untreated astrocytes (Fig. 3a). Steady-state hAPP levels, resulting from ongoing producing and processing activities, did not change following treatment with fAβ_25–35_ alone or with fAβ_25–35_ + NPS 2143 (Fig. 3a). Conversely, hAPP levels increased following exposure to fAβ_25–35_ + CMT versus fAβ_25–35_ alone, an effect that was amplified rather than counteracted by NPS 2143 (Fig. 3a).

Recent evidence indicates that promoting the delivery of hAPP to the plasma membrane or inhibiting the internalization of hAPP favours hAPP NAP^66^. Hence, we investigated the distribution of hAPP in cortical adult human astrocytes following fAβ_25–35_ ± CMT ± NPS 2143-treatment. We biotinylated proteins on the astrocyte cell surface and then assessed the amount of biotinylated hAPP at the plasma membrane, with the remaining non-biotinylated hAPP regarded as “intracellular” hAPP (see *Methods* for details).

Plasma membrane (i.e., biotinylated) hAPP levels (*i*) peaked at 48 h with fAβ_25–35_ alone; (*ii*) increased from 24 h to 72 h with NPS 2143 + fAβ_25–35_; (*iii*) did not change versus the controls with fAβ_25–35_ + CMT; and (*iv*) increased strongly and progressively with NPS 2143 + fAβ_25–35_ + CMT (Fig. 3b).

Simultaneously, intracellular hAPP levels (*i*) decreased steadily and similarly with fAβ_25–35_ ± NPS 2143; (*ii*) surged rapidly up to 24 h and then plateaued with fAβ_25–35_ + CMT; and (*iii*) after a 24-h delay, peaked at 48 h with NPS 2143 + fAβ_25–35_ + CMT and then began to decrease (Fig. 3c).

Thus, antagonizing Aβ•CaSR signalling intensified hAPP trafficking to the plasma membrane, an effect that was remarkably intensified by CMT.

### NPS 2143 drives the plasma membrane translocation of ADAM10 in astrocytes, reversing the sharp decline induced by fAβ_25–35_ ± CMT treatment

**Figure 4:**
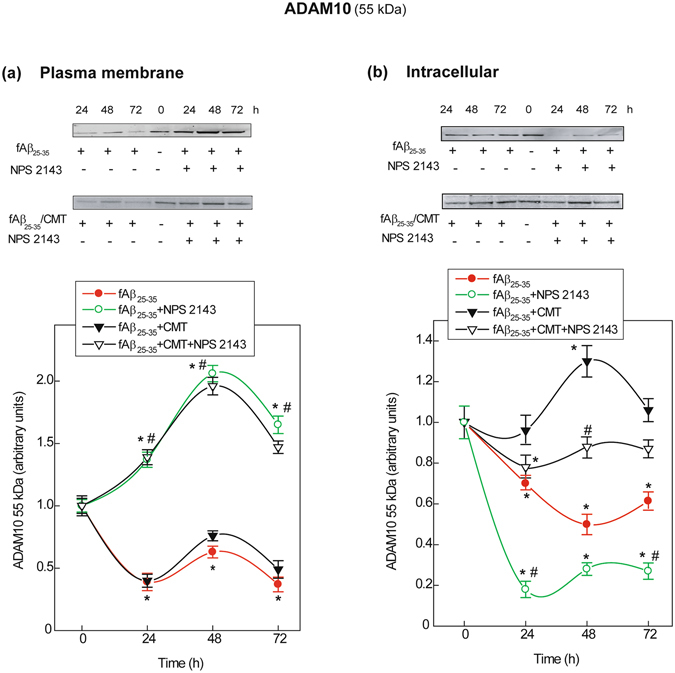
Changes in ADAM10 protein expression and subcellular distribution in human astrocytes. **(a**) *Top*. Typical immunoblots revealing changes in cell surface biotinylated ADAM10 levels (details in *Methods*) according to experimental treatments versus untreated controls. Blots have been cropped to size for clarity. *Bottom*. Densitometric evaluations of specific ADAM10 bands for each treatment and time point. Points in the curves are means ± SEM of 3 independent experiments, with 0-h (controls) values normalized as 1.0. *One-way ANOVA* analysis of (*i*) fAβ25–35 ± NPS2143 data set: *F* = 73.625, P < 0.001; (*ii*) fAβ_25–35_ + CMT ± NPS2143 data set: *F* = 55.591, P < 0.001; *Bonferroni t-test*: comparisons of each treated group *versus* 0-h group (controls): *P < 0.05; pair-wise comparisons between corresponding treatments ± NPS 2143 at each time point: ^**#**^P < 0.05. **(b**) *Top*. Characteristic immunoblots exposing changes in the levels of non-biotinylated intracellular ADAM10 according to the specific treatments versus untreated controls. In all cases blots have been cropped to size for clarity. *Bottom*. Densitometric evaluation of specific ADAM10 bands for each treatment and time point. Points in the curves are means ± SEM of 3 independent experiments, with 0-h values normalized as 1.0. *One-way ANOVA* analysis of (*i*) fAβ_25–35_ ± NPS2143 data set: *F* = 34.818, P < 0.001; (*ii*) fAβ_25–35_ + CMT ± NPS2143 data set: *F* = 10.938, P < 0.001; *Bonferroni t-test:* comparisons of each treated group *versus* 0-h (controls): *P < 0.05; pair-wise comparisons between corresponding treatments ± NPS 2143 at each time point: ^**#**^P < 0.05.

ADAM10 is the key α-secretase in hAPP physiological NAP^19, 25^. (Concerning the effects of the various treatments on ADAM17 α-secretase, see Fig. S2 in the Supplementary Information). Therefore, we assessed plasma membrane biotinylated and non-biotinylated (intracellular) ADAM10 (55-kDa active form) via immunoblotting (Fig. 4a,b).

#### Plasma membrane ADAM10

During the first 48 h, the level of plasma membrane biotinylated 55-kDa ADAM10 decreased rapidly in fAβ_25–35_ ± CMT-treated astrocytes (Fig. 4a). However, adding NPS 2143 to fAβ_25–35_ ± CMT increased plasma membrane ADAM10 levels up to 72 h.

#### Intracellular ADAM10

The levels of non-biotinylated (“intracellular”) 55 kDa ADAM10 greatly decreased versus the controls in fAβ_25–35_-treated astrocytes and decreased further when NPS 2143 was added to fAβ_25–35_ (Fig. 4b). Conversely, intracellular ADAM10 increased transiently after approximately 48 h in fAβ_25–35_ + CMT-exposed astrocytes, whereas NPS 2143 + Aβ_25–35_ + CMT marginally reduced intracellular ADAM10 levels.

### NPS 2143 ± CMT treatment increased the total ADAM10 specific activity in fAβ_25–35_-exposed human astrocytes

**Figure 5:**
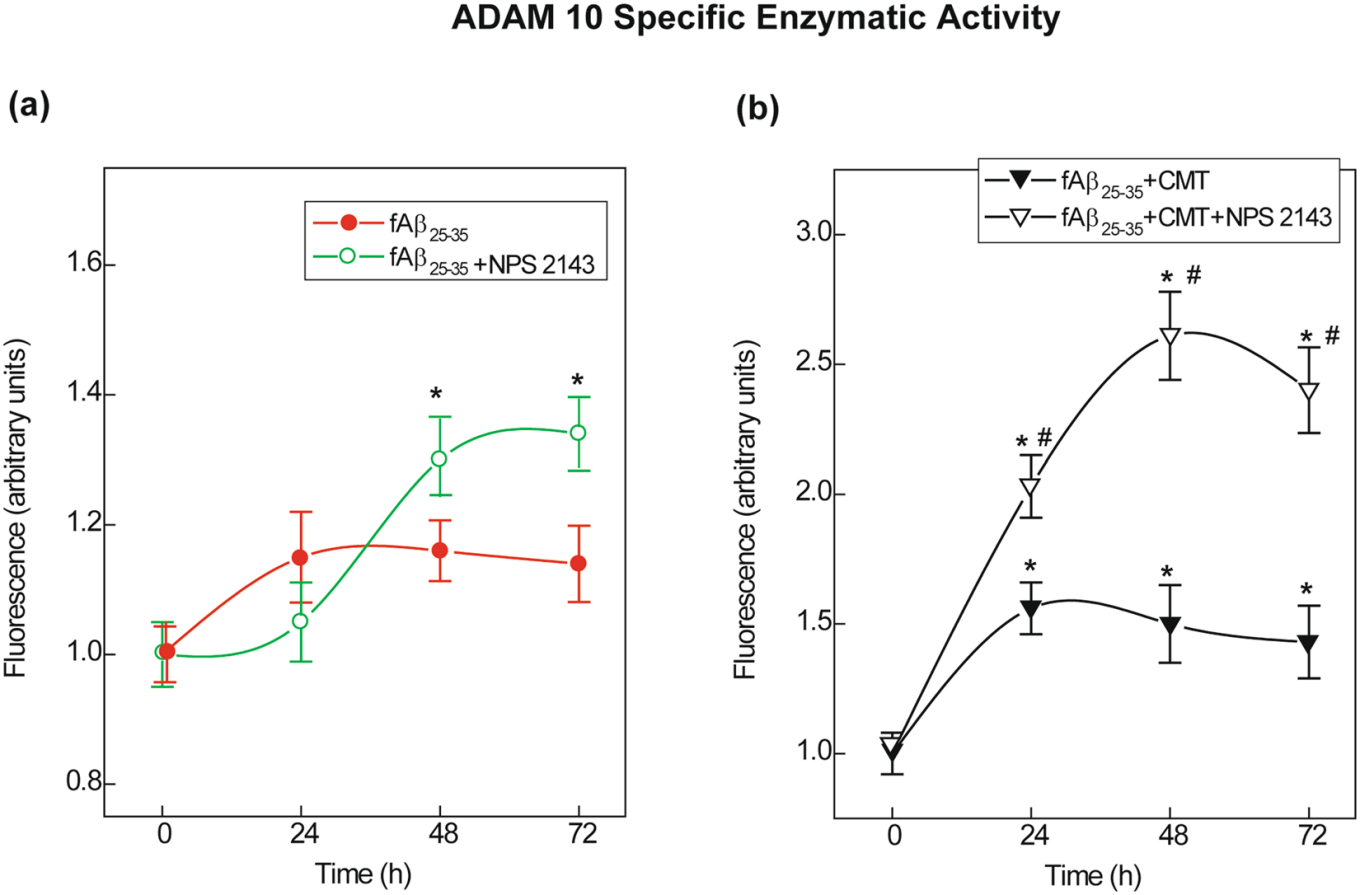
ADAM10 specific enzymatic activity alterations in human astrocyte protein lysates. **(a**) Changes in ADAM10 specific activity after an exposure to fAβ_25–35 _± NPS 2143. **(b**) Alterations in ADAM10 specific activity increases after treatment with fAβ_25–35_ + CMT ± NPS 2143. The specific activities in **A** and **B** were assayed as described in the *Methods* section. Points on the curves are means ± SEM of 3 independent experiments with 0-h values normalized as 1.0. *One-way ANOVA* analysis of*:* (*i*) fAβ_25–35_ ± NPS2143 data set: *F* = 7.923, P < 0.001; (*ii*) fAβ_25–35_ + CMT ± NPS 2143 data set: *F* = 74.248, P < 0.001; *Bonferroni t-test:* comparisons of each treated group versus 0-h (controls):*P < 0.05; pair-wise comparisons of corresponding treatments ± NPS 2143 at each time point: ^**#**^P < 0.05.

Based on the above results, we next assessed the specific activity of ADAM10 in total protein lysates (Fig. 5a,b), which (*i*) increased slightly with fAβ_25–35_ but substantially after 24 h with NPS 2143 + fAβ_25–35_; (*ii*) immediately increased and then remained steadily elevated with fAβ_25–35_ + CMT (cf. Fig. 5a); and (*iii*) surged rapidly with the addition of NPS 2143 to fAβ_25–35_ + CMT (Fig. 5b).

Therefore, fAβ_25–35_ alone decreased both plasma membrane and intracellular ADAM10 levels. Despite the presence of Aβ_25–35_ ± CMT, NPS 2143 increased the amount of active ADAM10 at the plasma membrane, i.e., where greater hAPP translocation was also occurring (cf. Fig. 2b). These NPS 2143-driven events restored neurotrophic sAPPα shedding. Additionally, although it did not hinder the NPS 2143-driven plasma membrane translocation of ADAM10, CMT strongly attenuated – through an undefined mechanism – the sharp decline in intracellular ADAM10 levels elicited by fAβ_25–35_ ± NPS 2143.

## Discussion

**Figure 6:**
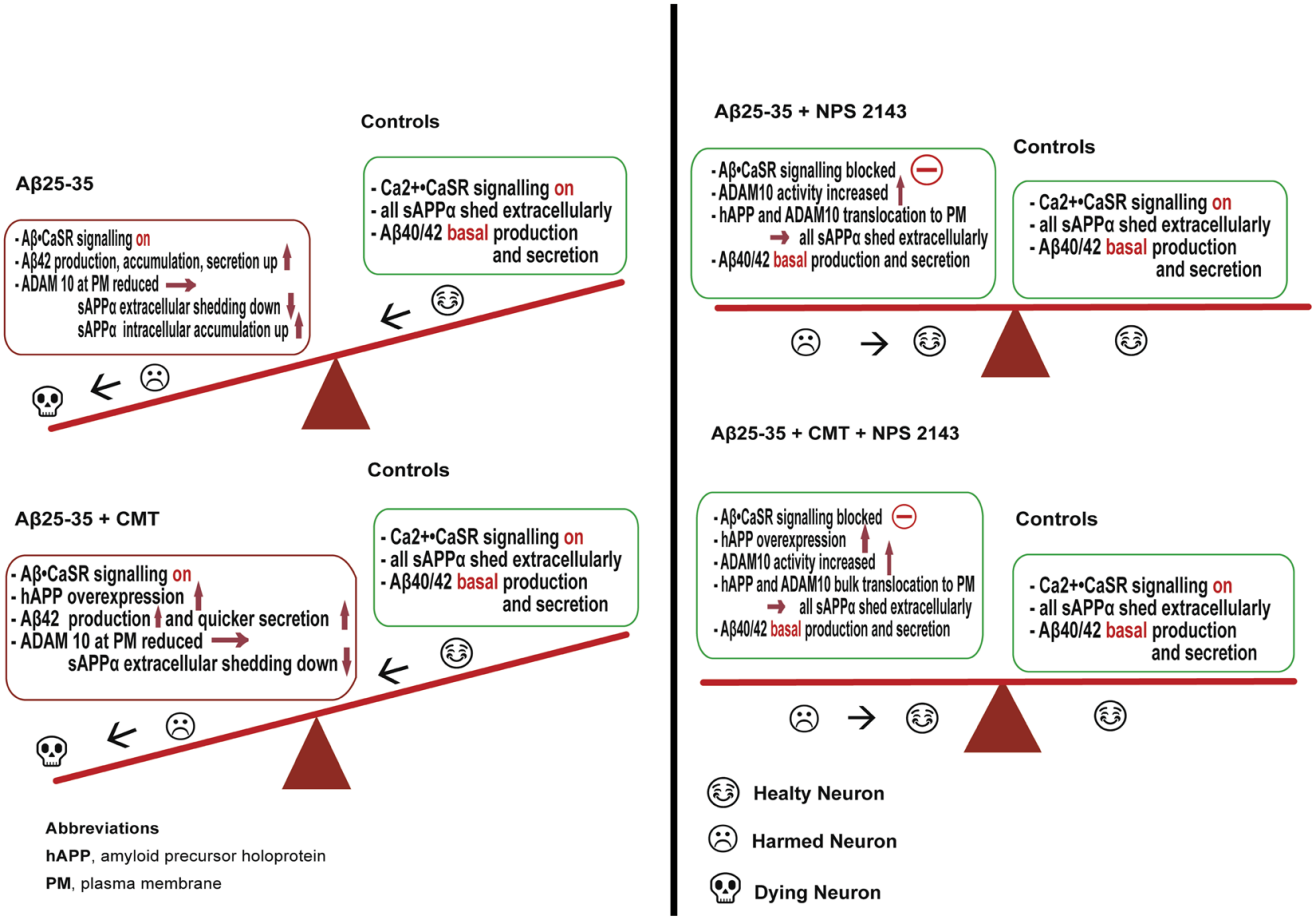
Mechanisms synopsis. Cartoon illustrating the concept that CaSR-dependent signalling can deeply affect the balance between hAPP NAP and AP and hence play a role in the promotion or prevention of AD development. *Left panel*. The main mechanisms by which Aβ_25–35_ alone (*top*) or +CMT (*bottom*) alters the hAPP physiological NAP in favour of AP. *Right panel*. The main mechanisms by which calcilytic NPS 2143 restores the hAPP NAP while hindering the AP in Aβ_25–35_-exposed adult human astrocytes either in absence (*top*) or in the presence of CMT (*bottom*). The emojis indicate what most likely happens to the neurons exposed to the various agents (human astrocytes survive). See the text for further details.

Currently, scant information is available about the mechanisms modulating hAPP proteolysis in cortical untransformed human neural cells. Our previous findings revealed that Aβ•CaSR signalling promotes the AP of hAPP, eliciting substantial increases in endogenous Aβ_42_ accumulation and secretion from adult human astrocytes and postnatal neurons^22–24^. Our present results show for the first time that a highly selective CaSR antagonist (calcilytic) rescues the physiological NAP of hAPP, maintaining neurotrophic and neuroprotective sAPPα shedding while fully suppressing pathological AP in Aβ-exposed human astrocytes and neurons, even in the presence of microglial CMT (Fig. 6). Although our results were obtained only in astrocytes, we previously demonstrated that the effects of Aβ•CaSR signalling and its antagonism by a calcilytic on Aβ_42_ metabolism are very similar in human neurons and astrocytes^22–24^. Hence, an extension of the present findings to neurons is feasible until experimentally proven. The concurrent suppression of NO overproduction and adenylate cyclase inhibition (Fig. S1) strengthens the view that NPS 2143 specifically antagonized Aβ•CaSR signalling in astrocytes.

Therefore, the present results prove for the first time that a calcilytic agent can effectively correct the balance of hAPP processing altered by Aβ•CaSR-signalling-mediated mechanisms in cortical human astrocytes (and likely neurons) (Fig. 6). The calcilytic rescues the α-secretase-mediated extracellular shedding of neurotrophic and neuroprotective sAPPα at the expense of the BACE1/β-secretase-mediated neurotoxic Aβ_42_ overrelease. The former would safeguard neuronal trophism, viability, and synaptic connections. The latter is inherently dangerous because overproduced and oversecreted soluble Aβ_42_/Aβ_42_-os and their insoluble fibrillar derivatives are endowed with a pernicious self-propagating potential. These peptides can react with CaSRs in adjacent and farther neurons and astrocytes, triggering self-spreading and self-perpetuating vicious waves of Aβ•CaSR signalling, and their consequent accumulation releases further Aβ_42_/Aβ_42_-os surpluses, which likely sustain LOAD progression^22–24^. Most remarkably, calcilytics can break such vicious cycles and hence stop Aβ_42_ oversecretion and intra-brain diffusion, thus safeguarding neuronal viability and function^22–24^. In addition, the calcilytic NPS 2143 elicits a robust downregulation of total CaSR levels in astrocytes, thereby inducing a lasting cell desensitization to exogenous Aβs•CaSR-driven noxious effects^22^.

Regarding the mechanisms of calcilytics, NPS 2143 notably (*i*) drives a substantial translocation of hAPP, as well as both the precursor (85 kDa; not shown) and active (55 kDa) forms of ADAM10 α-secretase, to the astrocyte plasma membrane; (*ii*) greatly increases the total ADAM10 α-secretase specific activity, particularly in the presence of CMT; (*iii*) maintains the intracellular levels of active ADAM10 (55 kDa) at or above basal values; and (*iv*) restores neurotrophic and neuroprotective sAPPα extracellular shedding close to untreated control levels while hindering the intracellular accumulation of sAPPα. Consequently, by concomitantly also suppressing any surplus Aβ_42_ release, NPS 2143 maintains the secreted Aβ_42_/sAPPα and Aβ_42_/Aβ_40_ ratios near controls levels.

As with other type-I transmembrane proteins, hAPP is synthesized in the endoplasmic reticulum (ER). Then, hAPP undergoes maturation (glycosylation) while migrating to the Golgi/TGN compartment, where it is mainly found in neurons^66^. Finally, hAPP reaches the plasma membrane via the constitutive secretory pathway, where it is inserted to be cleaved by ADAM10 (mainly) α-secretase, shedding the sAPPα ectodomain, which also occurs in a post-Golgi compartment^67^. In addition, through the recognition of its YENPTY motif and clathrin-coated pits, hAPP can be quickly endocytosed from the plasma membrane and trafficked back to the membrane, delivered through endosomes to the lysosomal system for proteolysis^66^ or alternatively cleaved by BACE1/β-secretase, shedding Aβs (the amyloidogenic pathway), particularly if retained in acidic late endosomes, the TGN or ER^67^. Thus, favouring the plasma membrane trafficking or retention of hAPP blocks Aβ production while enhancing sAPPα extracellular shedding via ADAM10 cleavage. hAPP trafficking is regulated by factors that promote Aβ generation, such as the SNX family (SNX17 and SNX33), dynamin I, and the RAB GTPase family (RAB1B, RAB6, RAB8, and RAB11)^68^. In addition, factors that regulating α−, β−, and γ-secretase trafficking are able to alter hAPP processing and, hence, impact the production of sAPPα or Aβs^68^. Further investigations will clarify the roles played by such factors in human astrocytes.

The activation of a number of cell surface receptors, e.g., muscarinic acetylcholine receptors, platelet-derived growth factor (PDGF) receptors, serotonin/5-hydroxytryptamine (5-HT_4_) receptors, and metabotropic glutamate receptors, reportedly exerts differential effects on hAPP AP or NAP^69^. Our findings add CaSRs to this group of receptors. These receptors activate various signalling pathways that regulate extracellular Aβ secretion and sAPPα shedding via changes in cytosolic [Ca^2+^]_i_, cAMP, inositol 1,4,5-triphosphate, small Rac GTPases, and in the activity of a number of protein kinases, including PKA, PKC, mitogen activated protein kinase kinase (MAPKK), extracellular signal-regulated kinase (ERK), phosphatidylinositol–3-kinase (PI3K), and Src tyrosine kinase^69^. Reduced cholesterol levels also heighten ADAM10 activity and hinder hAPP endocytosis, thus enhancing sAPPα shedding from cultured cells^69^. Similar effects can be obtained via ADAM10 overexpression^70^, pharmacological muscarinic activation^32^ or phorbol myristate acetate treatment in hAPP-transfected CHO cells^27^. Conversely, the AP of hAPP was favoured at the expense of sAPPα extracellular shedding following overexpression of BACE1/β-secretase^40^ or the Swedish mutant form of hAPP (SweAPP), which is linked to a familial EOFAD and is more effectively cleaved by BACE1/β-secretase within the TGN^69^. In addition, knocking down ADAM10^25^ and expressing a dominant-negative ADAM10 mutant in mice^70^ both increased hAPP AP.

ADAM family members belong to the metzincin superfamily and are typically synthesized as inactive precursors (zymogens)^71^. The proteolytic removal of a conserved cysteine switch in the prodomain is necessary to activate these zymogens^71^. Our findings indicate that cleavage by proprotein convertases (e.g., furin and PC7 in HEK293 cells^72^) into the 55-kDa ADAM10 active form occurs at the cell surface of human astrocytes rather than in late compartments of the secretory pathway. However, the complex mechanisms modulating α-secretase cleavage activity are not fully elucidated. ADAM10 is not the sole constitutive α-secretase in neurons^25, 73^. The present findings indicate that antagonizing Aβ·CaSR signalling with a calcilytic agent, in the absence but more effectively in the presence of CMT, increases the regulated ADAM10 α-secretase specific activity in adult human astrocytes. Treatment with NPS 2143 drives the plasma membrane translocation of both ADAM10 and hAPP in fAβ_25–35_ + CMT-exposed astrocytes. This finding reveals that Aβ·CaSR signalling alone restrains the vesicular transport of hAPP and ADAM10 to the plasma membrane, while raising hAPP intracellular levels and AP.

Although it increased ADAM10 α-secretase specific activity, CMT addition had little to no impact on daily and cumulative (i.e., over 72 h) extracellular sAPPα secretion. Only NPS 2143 addition restored sAPPα secretion to the levels of untreated astrocytes, showing the importance of blocking Aβ•CaSR signalling is in restoring hAPP NAP. Regarding the intracellular storage of sAPPα, which did not occur in the untreated astrocytes, CMT addition altered the kinetics but not the total amount stored in fAβ_25–35_-exposed astrocytes. As expected, NPS 2143 reduced most but not all) of the sAPPα storage caused by fAβ_25–35_ treatment, even in the presence of CMT. The reasons why these minor sAPPα fractions were retained regardless of NPS 2143 and CMT treatment are not currently understood. Intracellular sAPPα accumulation has also been observed in other cellular models, including cultured human thyroid cells^50^.

Increased cleavage of hAPP by α-secretase was previously suggested as a therapeutic approach to AD^32^. Our present results strengthen the role of calcilytics as prospective drugs for AD therapy (Fig. 6). In this regard, calcilytics benefits largely overcome the mild hyperparathyroidism they induce in humans, given that AD “*inexorably kills the patient cognitively several years before his/her actual physical demise*”^22–24^. Therefore, the negative consequences of calcilytics should prove negligible if clinical trials prove that they can halt AD development.

## Methods

### Cell cultures

Untransformed human adult astrocytes were isolated from anonymized surgical fragments of normal adult human temporal cortex (brain trauma leftovers) provided by several Neurosurgery Units after obtaining written informed consent from all the patients and/or their next-of-kin. Experimental use of isolated astrocytes was approved by the Ethical Committee of Verona University-Hospital Integrated Company. All human cells experiments were performed in accordance with the relevant guidelines and regulations of Verona University-Hospital Integrated Company. Cultures of astrocytes were set up, as previously described^22^, in a medium consisting of 89% (v/v) of a 1:1 mixture of DMEM and F-12 medium (Life Technologies Italia, Monza, Italy), 10% (v/v) heat-inactivated (at 56 °C for 30 min) foetal bovine serum (FBS; Life Technologies Italia) and 1% (v/v) of a penicillin–streptomycin solution (Lonza Milano, Italy). When the primary cultures reached 70% confluence (4 weeks), the cells were detached with 0.25% (w/v) trypsin and 0.02% (w/v) EDTA (Lonza) in Hanks BSS, split 1:4 and planted in new flasks. After the third subculture a homogeneous population of astrocytes obtained. In these pure cultures, the cells only expressed astrocyte-specific markers such as glial fibrillary acid protein (GFAP) and glutamine synthase (GS). None of the cells expressed neuronal (enolase), oligodendrocytes (galactocerebroside), microglia (CD-68), or endothelial cells (factor VIII) markers. These astrocytes proliferated quite slowly in serum-enriched DMEM (Life Technologies Italia) medium and were by now phenotypically “locked-in”.

### Aβ peptides

Aβ_25–35_ (Bachem), a known Aβ_1–42_ proxy^22^, was dissolved at 1.5 mM in PBS. Fibrillogenesis by Aβ_25–35_ was checked *via* thioflavin-T tests before experimental use. The reversemer peptide Aβ_35–25_ (Bachem) was dissolved in the same way as Aβ_25–35_, but did not form fibrils and when given to the cultures was totally ineffective (not shown).

### Experimental protocol

Since astrocytes do not actively divide in the adult human brain, we employed them once they had reached mitotic quiescence. At experimental “0 h”, culture flasks served partly as untreated controls receiving a change of fresh medium and partly received fresh medium with 20 µM of either fibrillar (f)Aβ_25–35_ or reversemer Aβ_35–25_ added. This dose of the fAβs had been found to be ideal in previous studies^22–24^. Part of the treated cultures received 20 µM of fAβ_25–35_ once (at 0 h) plus a cytokine mixture trio (CMT), that is IL-1β (20 ng mL^−1^), TNF-α (20 ng mL^−1^), and IFN-γ (70 ng mL^−1^) (all from PeproTech, London, England). A second and a third CMT bolus was added at 24-h and 48-h. The CaSR allosteric antagonist (calcilytic) NPS 2143 HCl (2-chloro-6-[(2 R)–3-1,1-dimethyl-2-(2-naphtyl) -ethylamino-2-hydroxy-propoxy]-benzonitrile HCl; Tocris Bioscience, UK)^54^ was dissolved in DMSO and next diluted in the growth medium at a final concentration of 100 nM. At experimental “0-h”, “24-h”, and “48-h” part of the astrocyte cultures were exposed for 30 min to NPS 2143 dissolved in fresh medium. Next, the NPS 2143-containing medium was removed and fresh (at 0.5-h) medium or the previously astrocyte-conditioned (at 24.5 and 48.5-h) media were added again to the cultures. Cultures and cell-conditioned media were sampled at 24 hourly intervals. Phosphoramidon (10 μM; Sigma), an inhibitor of thermolysin and other proteases, was added to the media at “0-h” experimental time.

### Western immunoblotting (WB)

At selected time points, control and treated astrocytes were scraped into cold PBS, sedimented at 200 × *g* for 10 min, and homogenized in T–PER™ tissue protein extraction reagent (Thermo Scientific, Rockford, USA) containing complete EDTA–free protease inhibitor cocktail (Roche, Milan). Equal amounts (10–30 µg) of protein from the samples were loaded on NuPAGE Novex 4–12% Bis–Tris polyacrylamide gel (Life Technologies Italia) and next blotted onto nitrocellulose membranes (0.2 µm) by means of iBlot^TM^ Dry Blotting System (Life Technologies Italia). The membranes were probed with: (*i*) rabbit polyclonal anti-Amyloid Precursor Protein (APP), C-Terminal (751–770) diluted 1:2000 (Calbiochem; Merck, Darmastadt, Germany); (*ii*) mouse monoclonal 2B3 antibody anti-sAPPα human C-terminal peptide (IBL International GmbH, Hamburg, Germany), diluted at 2.0 µg mL^−1^; (*iii*) mouse monoclonal antibody anti-ADAM10 (A3, Santa Cruz Biotechnology, Germany) used at 1.0 µg mL^−1^; (*iv*) mouse monoclonal antibody anti-Aβ_42_ (8G7, Acris Antibodies GmbH, Germany) used at 1.0 µg mL^−1^; and (*v*) goat polyclonal antibody anti-lamin B (Santa Cruz Biotechnology) which served to assess loading controls used at 1.0 µg mL^−1^. The integrated intensities of the bands specific for each protein of interest were assessed using the Sigmagel™ software package (Jandel Corp., Erkrath, Germany).

### Biotinylation and isolation of astrocytes’ plasmalemmal proteins

The Pierce^TM^ Cell Surface Protein Isolation Kit (Thermo Scientific) served to biotinylate and isolate cell surface proteins. According to the supplier’s procedure the cell culture media were removed and astrocytes were washed twice with ice-cold PBS followed by incubation with 0.25 mg mL^−1^ Sulfo-NHS-SS-Biotin in ice-cold PBS on a rocking platform for 30 minutes at 4 °C. The biotinylation reaction was quenched by adding 500 μl of the provided Quenching Solution (Pierce). Astrocytes were harvested by gentle scraping and pelleted by centrifugation at 500 × *g* for 5 minutes at 4 °C. After washing with TBS astrocyte pellets were lysed using the provided Lysis Buffer (Pierce) containing a protease inhibitor cocktail (Roche) for 30 minutes on ice with intermittent vortexing. To get rid of cell remnants, the lysates were centrifuged at 10,000 × *g* for 2 minutes at 4 °C. To purify biotinylated proteins on Immobilized NeutrAvidin Gel, the clarified supernatant was incubated for 1-h at room temperature (RT) to allow the biotinylated proteins to bind to the NeutrAvidin Gel. The unbound proteins, representing the intracellular fraction, were collected by centrifugation of the column at 1,000 × *g* for 2 minutes. Any remaining unbound proteins were removed by washing thrice with Wash Buffer (Pierce). Finally, the biotinylated surface proteins were eluted from the biotin-NeutrAvidin Gel by incubation with 400 µL of the SDS-PAGE Sample Buffer containing 50 nM DTT for 1-h at RT in the end-over-end tumbler, and were collected by column centrifugation at 1,000 × *g* for 2 minutes.

### Assays of α-secretases specific activities

The ADAM10 and ADAM17 enzymatic activities were assayed by means of fluorescent methods using EnSens^TM^ ADAM10 and EnSens^TM^ ADAM17 activity detection kits (Enzium, Inc., Philadelphia, USA) in the cell lysates. Despite the highly-overlapping substrate specificities of ADAM10 and ADAM17, EnSens^TM^ substrates are able to differentiate between the two enzymes. Astrocytes’ lysates (20 μg) were incubated with the fluorogenic EnSens^TM^ ADAM10 and EnSens^TM^ ADAM17 substrates, respectively for 1-h at RT, protected from light according to the supplier’s protocol. The fluorescence was recorded at excitation and emission wavelengths of 625–635 nm and 655–665 nm, respectively. The results were expressed as specific activity (means ± SEMs of ΔF µg^−1^ protein pertaining to each experimental group).

### Enzyme-linked immunoassays (ELISAs) of Aβ_42_, Aβ_40_, and sAPPα released into in cell-conditioned growth media

Quantifications of Aβ_42_, and Aβ_40_ and sAPPα were carried out by means of specific Aβ_42_, and Aβ_40_ Human/Rat High-Sensitive ELISA Kits (both from Wako, Japan) as previously described^22^ and by means of specific Human sAPPα High-Sensitive ELISA Kit (from IBL International). Briefly, the astrocytes conditioned media samples were added with a protease inhibitor cocktail (Roche) and centrifuged for 10 minutes at 13,000 rpm to remove any cellular debris. Supernatants were tested in triplicate according to the manufacturer protocol.

### Statistical analysis

The data were analyzed using Sigma Stat 3.5™ Advisory Statistics for Scientists (Systat Software). For immunoblotting, bands’ densitometric data were normalized to matching loading control (lamin B1) bands and next analyzed by *one–way* *ANOVA*. When the *ANOVA*’s upshot was significant (P < 0.05), Bonferroni *t*-test, was used for all pairwise comparisons and multiple comparisons versus 0-h (untreated) control values. Null hypotheses were rejected when P > 0.05.

## Electronic supplementary material

Supplementary Figures
