# Supplementary material for: Calcium-Sensing Receptor Antagonist NPS 2143 Restores Amyloid Precursor Protein Physiological Non-Amyloidogenic Processing in Aβ-Exposed Adult Human Astrocytes
Source: Sci Rep. 2017 Apr 28;7:1277. doi: 10.1038/s41598-017-01215-3 (PMC5430644; doi:10.1038/s41598-017-01215-3)
Supplement: Supplementary file 1 — Supplementary Figures [file 41598_2017_1215_MOESM1_ESM.pdf]

CALCIUM-SENSING RECEPTOR ANTAGONIST NPS 2143 RESTORES AMYLOID PRECURSOR PROTEIN PHYSIOLOGICAL NON-AMYLOIDOGENIC PROCESSING IN Aβ-EXPOSED ADULT HUMAN ASTROCYTES

Anna Chiarini[1], Ubaldo Armato[1], Daisong Liu[2], Ilaria Dal Prà[1]

[1]*Human Histology & Embryology Unit, Medical School, University of Verona, Verona, Venetia, I-37134, Italy*

[2]*The Third Xiangya Hospital of Central South University, Department of Plastic Surgery, Changsha, Hunan, China*

Supplementary Results and Discussion

NPS 2143 fully quells any NO surplus release from fAβ_{25-35}+CMT-treated astrocytes.

It is well established that the neurotoxic upshots of Aβ•CaSR signaling do not exclusively entail the boosting of hAPP proteolytic processing along the pathological amyloidogeni**c** pathway[1,2,3]. Even the separate exposure to either Aβ_{25-35} or Aβ_{1-42} or to CMT elicit the release of NO surpluses[1,4,5]. Therefore, we assessed how NPS 2143 would affect NO release from fAβ_{25-35}+CMT-treated astrocytes.

NO release increased by 12.4-fold versus controls by 72-h after the exposure to fAβ_{25-35}+CMT-treated astrocytes (Supplementary **Fig. S1A**). Daily treatments with NPS 2143 brought back the amount of NO astrocytes released to the level of untreated controls (Supplementary **Fig. S1A**).

Thus, calcilytics can also effectively avoid any additional cytotoxic damage otherwise elicited by NO overreleased from fA$\beta_{25\text{-}35}$+CMT-treated astrocytes via an activated Aβ•CaSR and MEK/ERK signaling.

NPS 2143 fully hinders the fall of intracellular cyclic AMP (cAMP) levels in fA$\beta_{25\text{–}35}$+CMT in astrocytes.

Via various G proteins CaSR's signaling activates panoply of cellular signaling pathways, one of which inhibits adenylate cyclase activity and hence cAMP synthesis from ATP[6,7]. Conversely, by antagonizing Aβ•CaSR's signaling calcilytics boost adenylate cyclase's production of cAMP[8]. Reportedly, the adenylate cyclase/cAMP pathway modulates ADAM10 activity and hAPP processing in neurons[9]. Therefore, to assess whether the adenylate cyclase/cAMP cascade might modulate sAPPα shedding from human astrocytes too, we had to assess the cAMP level changes caused by each experimental treatment. As our results revealed, *(i)* human astrocytes basally produced discrete amounts of cAMP, of which 80% were intracellular and 20% were released into the medium; *(ii)* fA$\beta_{25\text{–}35}$+CMT treatment reduced total cAMP levels after 30-min; and *(iii)* in the fA$\beta_{25\text{–}35}$+CMT-exposed astrocytes a 30-min pre-treatment with NPS 2143 kept discretely above untreated control levels the intracellular and total cAMP levels assayed 30-min later (Supplementary **Fig. S1B**). During the same time, the cAMP levels in the supernatants of the experimental groups *(ii)* and *(iii)* did not significantly change versus control or group *(i)* levels (Supplementary **Fig. S1B**). In keeping with the findings of Yarova *et al.*[8], our preliminary results further indicate that fA$\beta_{25\text{–}35}$ activates CaSR signaling which, by inhibiting adenylate cyclase activity, quickly reduces intracellular and total cAMP levels in adult human astrocytes cultures. Moreover, they show that calcilytic NPS 2143 specifically suppresses the inhibitory effects of fA$\beta_{25\text{–}35}$•CaSR signaling on adenylate cyclase activity keeping cAMP at discretely higher levels than controls' range.

NPS 2143 does not impact on ADAM17 α-secretase specific activity in total protein lysates of f$A\beta_{25-35}$±CMT-exposed adult human astrocytes

We also investigated the specific enzymatic activity of ADAM 17, the second putative main α-secretase involved in the non-amyloidogenic processing of hAPP[10,11,12,13] under the several experimental conditions used in this work. As our observations showed, the specific enzymatic activity of ADAM17 discretely increased (by 48-h, +32%, P<0.05 versus controls) in total protein lysates of f$A\beta_{25-35}$ alone-exposed astrocytes (Supplementary **Fig. S2A**). Adding NPS 2143 had no effect at all on such increase and its kinetics (Supplementary **Fig. S2A**). Yet, ADAM17 specific activity surged much more (by 24-h, +76%; by 48-h, +124%; and by 72-h, +82%; P<0.001 in all instances versus controls) in the lysates of f$A\beta_{25-35}$+CMT treated astrocytes (Supplementary F**ig. S2B**). Then again, NPS 2143's addition did affect this increase in ADAM17 specific activity further in the lysates of astrocytes exposed to f$A\beta_{25-35}$±CMT (Supplementary **Fig. S2B**). Therefore, f$A\beta_{25-35}$±CMT amplified ADAM17 α-secretase specific activities, yet the ADAM17's surges, at variance with ADAM10's ones, were calcilytic-insensitive and hence not affected by Aβ•CaSR signaling, although other receptors for Aβs and CMT might have played their roles.

Materials and Methods

Assay of NO released into cell-conditioned growth media

The total NO_2^- concentration was determined in 50 μL samples of the culture supernatants as previously described[4] according to the fluorometric method based upon the reaction of NO_2^- with 2,3-diamino-naphthalene (DAN; Sigma-Aldrich) to form the fluorescent 1-(H)-naphthotriazole[14].

Enzyme-linked immunosorbant assays (ELISAs) of intracellular and secreted cAMP

Astrocytes were seeded into 24-well Multiwell Plate BD Primaria™ (BD Biosciences, USA) at a density of 40,000 cells/well. At experimental "0-h", some of such cultures served as untreated controls while others had their medium added for 30 minutes with 20 μM of f$A\beta_{25-35}$+CMT. Other cultures were first exposed to calcilytic NPS 2143 100 nM for 30 min and next underwent the same

30 min treatment with f$A\beta_{25-35}$+CMT prior to proteins and media harvesting. cAMP measurements were performed using DetectX Direct Cyclic AMP ELISA kit (Arbor Assays, USA) following the acetylated protocol enclosed to the manufacturer's instructions.

Statistical analysis

The data were analyzed using Sigma Stat 3.5™ Advisory Statistics for Scientists (Systat Software) using *one–way ANOVA*. When ANOVA's upshots of were significant ($P<0.05$), *Post-hoc Bonferroni's test* was used for comparisons versus 0-h (untreated) controls or between treated groups at the devised time points.

References

1. Armato, U. *et al*. Calcium-sensing receptor antagonist (calcilytic) NPS 2143 specifically blocks the increased secretion of endogenous Aβ42 prompted by exogenous fibrillary or soluble Aβ25-35 in human cortical astrocytes and neurons -Therapeutic relevance to Alzheimer's disease. *Biochim Biophys Acta - Mol Basis Dis* **1832**, 1634–1652 (2013).
2. Dal Prà, I. *et al.* Do astrocytes collaborate with neurons in spreading the "infectious" Aβ and Tau drivers of Alzheimer's disease? *Neuroscientist* **21**, 9-29 (2015).
3. Chiarini, A., Armato, U., Liu, D., Dal Prà, I. Calcium-sensing receptors of human neural cells play crucial roles in Alzheimer's disease. *Front Physiol* **7**, 134 (2016).
4. Chiarini, A., Dal Pra, I., Menapace, L., Pacchiana, R., Whitfield, J. F., Armato, U. Soluble amyloid beta-peptide and myelin basic protein strongly stimulate, alone and in synergism with combined proinflammatory cytokines, the expression of functional nitric oxide synthase-2 in normal adult human astrocytes. *Int J Mol Med* **16**, 801-807 (2005).
5. Dal Prà, I., Chiarini, A., Nemeth, E. F., Armato, U., Whitfield, J. F. Roles of the Ca2+-sensing receptor (CaSR) in the expression of inducible NOS (nitric oxide synthase)-2 and its BH4 (tetrahydrobiopterin)-dependent activation in cytokine-stimulated adult human astrocytes. *J Cell Biochem* **96**, 428-438 (2005).
6. Brown, E. M., MacLeod, R. J. Extracellular calcium sensing and extracellular calcium signaling. *Physiol Rev* **81**, 239-296 (2001).
7. Maiti, A., Hait, N. C., Beckman, M. J. Extracellular calcium-sensing receptor activation induces vitamin D receptor levels in proximal kidney HK-2G cells by a mechanism that requires phosphorylation of p38alpha MAPK. *J Biol Chem* **283**, 175-183 (2008).
8. Yarova, P. L. *et al.* Calcium-sensing receptor antagonists abrogate airway hyperresponsiveness and inflammation in allergic asthma. *Sci Transl Med* **7**, 284ra60 (2015).
9. Canepa, E. *et al.* Cyclic adenosine monophosphate as an endogenous modulator of the amyloid-β precursor protein metabolism. *IUBMB Life* **65**, 127-133 (2013).

10. Kojro, E., Fahrenholz, F. The non-amyloidogenic pathway: structure and function of alpha-secretases. *Subcell Biochem* **38**, 105-127 (2005).

11. Sastre, M., Walter, J., Gentleman, S. M. Interactions between APP secretases and inflammatory mediators. *J Neuroinflammation* **5**, 25 (2008).

12. Deuss, M., Reiss, K., Hartmann, D. Part-time alpha-secretases: the functional biology of ADAM 9, 10 and 17. *Curr Alzheimer Res* **5**, 187-201 (2008).

13. Bandyopadhyay, S., Goldstein, L. E., Lahiri, D. K., Rogers, J. T. Role of the APP non-amyloidogenic signaling pathway and targeting alpha-secretase as an alternative drug target for treatment of Alzheimer's disease. *Curr Med Chem* **14**, 2848-2864 (2007).

14. Misko, T. P., Schilling, R. J., Salvemini, D., Moore, W. M., Currie, M. G. A fluorometric assay for the measurement of nitrite in biological samples. *Anal Biochem* **214**, 11-16 (1993).

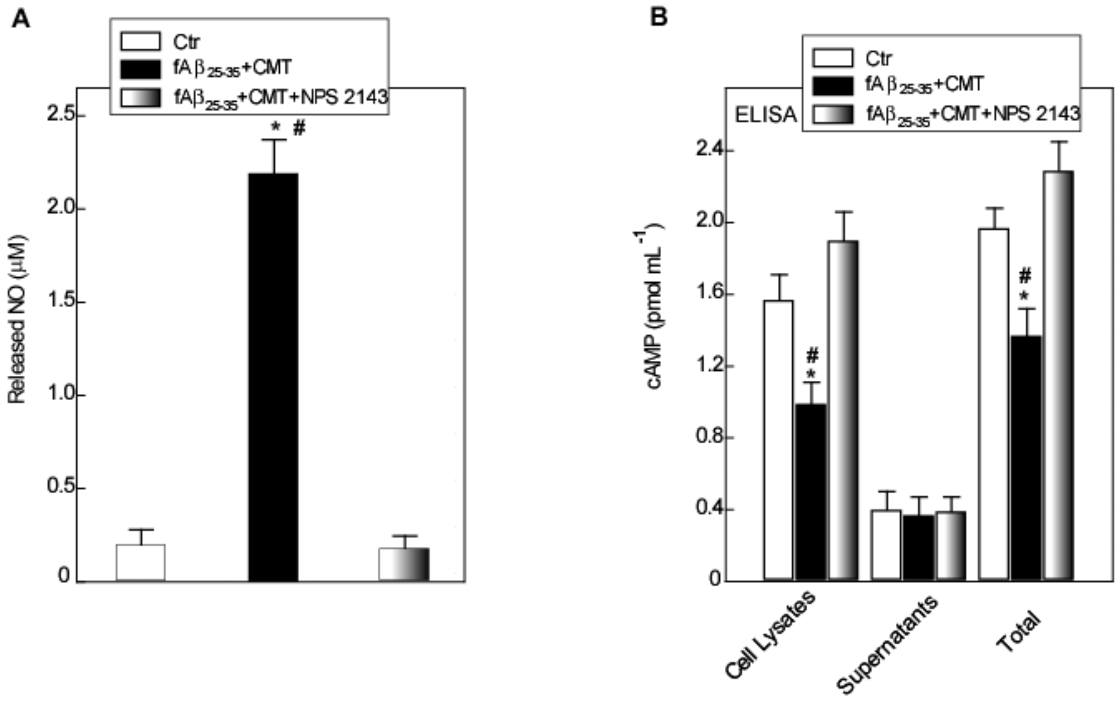

Supplementary Figure S1. NPS 2143 suppresses surplus NO release and secreted and intracellular cAMP fall in fA$\beta_{25\text{–}35}$**+CMT-treated astrocytes**

A The release of NO by adult human astrocytes is strongly increased by fA$\beta_{25\text{–}35}$+CMT treatment but is totally prevented by the concurrent addition of calcilytic NPS 2143. NO levels in the growth media were assessed as described in the *Materials and Methods* section. Bars are means ± SEMs of 5 independent experiments. *One-way ANOVA analysis*: F= 472.561, P<0.001; *Post-hoc Bonferroni test:* comparisons of each treated group versus 0-h (controls): *P<0.05; pairwise comparisons of fA$\beta_{25\text{–}35}$+CMT-treated group versus NPS 2143-added group: $^{\#}$P<0.05.

B Quantification of intracellular, secreted and total cAMP levels by specific ELISA analysis. Adult human astrocytes basally produce discrete amounts of cAMP but fA$\beta_{25\text{–}35}$+CMT treatment reduces both intracellular and total cAMP levels versus control ones. Adding NPS 2143 to fA$\beta_{25\text{–}35}$+CMT-exposed astrocytes keeps intracellular and total cAMP levels discretely above untreated control levels. Bars are means ± SEMs of 5 independent experiments. *One-way ANOVA analysis* of the: *(i)* intracellular cAMP data set: F= 17.653, P<0.003; *(ii)* total cAMP data set: F= 20.536, P<0.002; *Post-hoc Bonferroni test*: comparisons of each treated group versus 0-h (controls): *P<0.05; pairwise comparison of fA$\beta_{25\text{–}35}$+CMT-treated group versus NPS 2143-added group: $^{\#}$P<0.05.

ADAM 17 Specific Enzymatic Activity

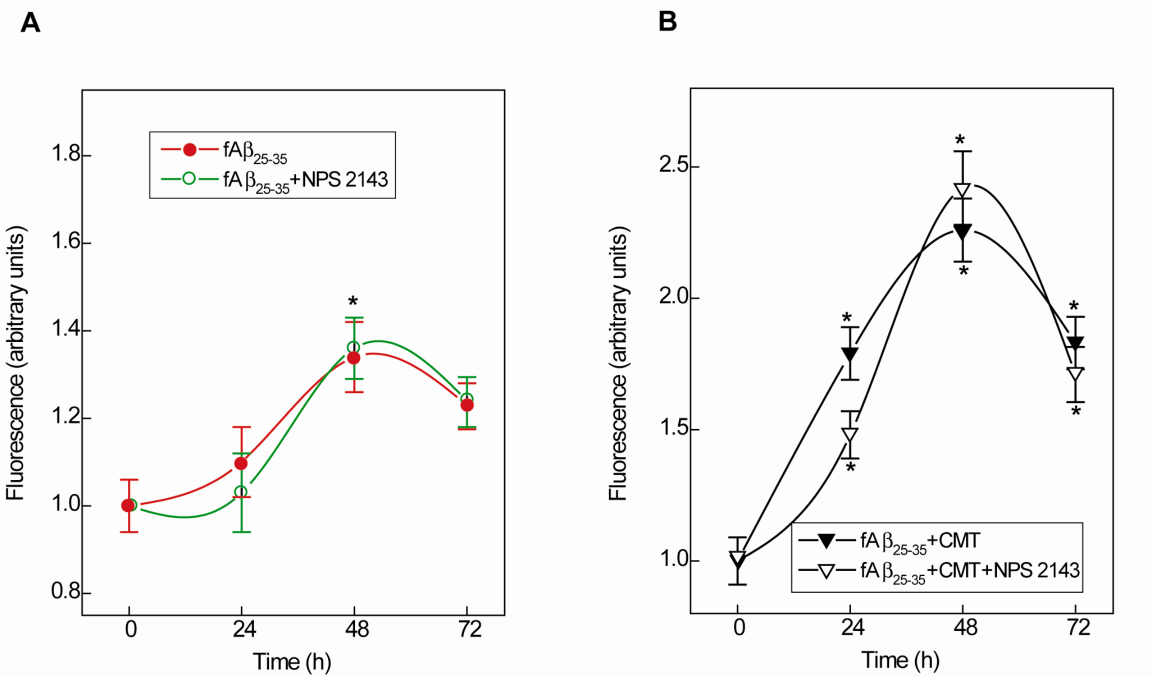

Supplementary Figure S2. ADAM17 specific enzymatic activity levels increase after an exposure to several treatments but NPS 2143 does not affect them.

A ADAM17 specific activity peaks after an exposure of 48-h to fA$\beta_{25\text{–}35}$ and adding NPS 2143 does not modify such a surge in adult human astrocytes.

B Under fA$\beta_{25\text{–}35}$+CMT-treatment ADAM17 specific activity further increases versus fA$\beta_{25\text{–}35}$ alone-treatment and NPS 2143's addition does not affect this increase.

The specific activities in **A** and **B**, were assayed as described in the *Materials and Methods* section. Points on the curves are means ± SEMs of 3 separate experiments with 0-h values normalized as 1.0. *One-way ANOVA analysis* of the: *(i)* fA$\beta_{25\text{–}35}$±NPS2143 ADAM17 set: F= 6.989, P<0.001; (*ii)* fA$\beta_{25\text{–}35}$+CMT±NPS2143 set: F= 41.478, P<0.001; *Post-hoc Bonferroni* test: comparisons of each treated group versus 0-h (controls): *, P<0.05.
